# Atypical Processing of Gaze Cues and Faces Explains Comorbidity between Autism Spectrum Disorder (ASD) and Attention Deficit/Hyperactivity Disorder (ADHD)

**DOI:** 10.1007/s10803-017-3078-4

**Published:** 2017-03-02

**Authors:** Madeleine J. Groom, Puja Kochhar, Antonia Hamilton, Elizabeth B. Liddle, Marina Simeou, Chris Hollis

**Affiliations:** 1 Division of Psychiatry and Applied Psychology, School of Medicine, University of Nottingham, Innovation Park, Triumph Road, Nottingham, NG7 2TU UK; 2 Derbyshire Healthcare NHS Foundation Trust, Temple House, Mill Hill Lane, Derby, DE23 6SA UK; 3 Institute of Cognitive Neuroscience, University College London, London, UK; 4 School of Psychology, University of Nottingham, Nottingham, UK

**Keywords:** ASD, ADHD, ERPs, Comorbidity, Gaze cueing, Face processing

## Abstract

This study investigated the neurobiological basis of comorbidity between autism spectrum disorder (ASD) and attention deficit/hyperactivity disorder (ADHD). We compared children with ASD, ADHD or ADHD+ASD and typically developing controls (CTRL) on behavioural and electrophysiological correlates of gaze cue and face processing. We measured effects of ASD, ADHD and their interaction on the EDAN, an ERP marker of orienting visual attention towards a spatially cued location and the N170, a right-hemisphere lateralised ERP linked to face processing. We identified atypical gaze cue and face processing in children with ASD and ADHD+ASD compared with the ADHD and CTRL groups. The findings indicate a neurobiological basis for the presence of comorbid ASD symptoms in ADHD. Further research using larger samples is needed.

## Introduction

Autism spectrum disorder (ASD) and attention deficit hyperactivity disorder (ADHD) are neurodevelopmental disorders that have significant, adverse effects on cognitive and socio-emotional development. The two are diagnosed as distinct conditions: ASD is defined by significant impairments in reciprocal social interaction and communicative function and restricted, repetitive behaviours and interests while ADHD is defined by developmentally inappropriate and functionally impairing levels of hyperactivity, impulsivity and inattention (American Psychiatric Association 2013; WHO 1992). In practice however, the two often co-occur. Prevalence of ADHD in children with ASD diagnosis is estimated between 37 and 85% in clinic cases (reviewed in Leitner 2014). Similarly, ASD symptoms are significantly higher in children with ADHD than in the general population (Kochhar et al. 2011; Mulligan et al. 2009). Recently, this diagnostic co-occurrence was formally recognised in the DSM-5 (American Psychiatric Association 2013) so that these diagnoses are no longer mutually exclusive. Despite this important change, still relatively little is known about the neurobiological basis of overlap between these conditions. There is evidence of shared genetic risk factors (Martin et al. 2014), but the mechanisms through which these shared risk factors manifest in the comorbid phenotype are unknown. Measuring cognitive markers of brain systems that are implicated in one or both conditions provides a useful tool for investigating the basis of comorbidity and may help distinguish between true overlap and ‘phenocopy’ (where the symptoms of one disorder mimic those of another, but arise from different pathways) (Rommelse et al. 2011).

Orienting visual attention in the direction of another’s eye gaze is a feature of social cognition that develops early in infancy and is a precursor to higher level abilities such as joint attention and theory of mind (Johnson et al. 2005). Experimental work using an adapted version of the Posner visuo-spatial cueing paradigm (Posner 1980) has shown that in healthy individuals, gaze cues elicit involuntary, automatic shifts of visuospatial attention towards a gazed at location (Frischen et al. 2007). In ASD participants, gaze cues often produce the same behavioural effect (reviewed in Nation and Penny 2008) but it has been suggested that unlike typically developing individuals, participants with ASD do not orient attention reflexively to gaze cues. For instance, one study measuring Inhibition of Return (IoR) (Marotta et al. 2013), a phenomenon thought to reflect the extent of reflexive orienting induced by a directional stimulus, found a robust IoR to gaze cues in healthy subjects but not ASD. This suggests that gaze cues do not elicit reflexive shifts of attention in those with ASD.

Neuroimaging studies provide further evidence of atypical processing of gaze cues in children and adults with ASD compared with typically developing individuals across a range of gaze processing tasks (Davies et al. 2011; Greene et al. 2011; Pelphrey et al. 2002; Pitskel et al. 2011; Vaidya et al. 2011). Electrophysiological studies have further shown reduced amplitude and altered topography of electrophysiological indices of face and gaze processing in those with (Senju et al. 2005; Tye et al. 2013) and at risk of (Elsabbagh et al. 2009) ASD, as well as atypical patterns of neural communication between brain regions while viewing gaze stimuli (Lajiness-O’Neill et al. 2014). Altogether, these findings suggest that orienting of attention to gaze cues is altered in ASD and this may contribute to broader social cognitive impairments identified in these individuals (Nomi and Uddin 2015). Indeed, it has recently been shown that atypical processing of gaze cues in infants at risk of autism is predictive of the quality of parent–child interactions (Elsabbagh et al. 2015) and ASD diagnosis at age 36 months (Elsabbagh et al. 2012). The neural processing of gaze cues may therefore be one useful marker with which to investigate the overlap between ASD and ADHD.

One previous study reported reduced orienting to gaze cues in children with ADHD (Marotta et al. 2014). This study further showed that this effect was specific to gaze cues when compared with directional arrow cues and peripheral sudden onset cues, suggesting that the effects could not be explained by general attentional mechanisms. However, in this study there was no comparison with ASD and although the authors excluded comorbid ASD, they did not measure the effect of sub-threshold ASD traits which may be significantly elevated in children with ADHD, even after excluding those who meet risk criteria for a possible ASD diagnosis (Kochhar et al. 2011). Further work is therefore needed to determine whether orienting of attention to gaze cues is altered in ADHD and whether any impairments are accounted for by co-occurring ASD. More broadly, this will potentially point to brain systems that explain the overlap between ADHD and ASD.

In the present study behavioural and electrophysiological correlates of attentional orienting to directional gaze and arrow cues were compared between children with ASD or ADHD aged 8–15 years and typically developing children. The ASD/ADHD sample included children who met diagnostic criteria for both. EEG data were recorded during task performance and event-related potentials (ERPs) were derived. ERPs provide excellent temporal resolution to measure very rapid, early, covert shifts of attention that are not observable in overt performance measures. One such ERP, the Early Directing Attention Negativity (EDAN) is larger in amplitude over the hemisphere contralateral to an attended visual field (VF). For example, when a cue directs attention towards the observer’s left VF, the EDAN will show larger amplitude over right than left hemisphere electrodes. This topographical effects indicates that the EDAN reflects the automatic orienting of attention to a spatial location (Jongen et al. 2007). Several studies have reported a lack of EDAN for gaze cues (Brignani et al. 2009; Hietanen et al. 2008; Holmes et al. 2010). However, two of these studies used schematic stimuli instead of real face images (Brignani et al. 2009; Holmes et al. 2010) and one study did not measure the EDAN occipitally, only parietally (Brignani et al. 2009). The other study manipulated the emotional expression of the face (Holmes et al. 2010), potentially weakening the EDAN effect or introducing other electrophysiological components that obscured it. In the present study we used photographic faces with a neutral expression, consistent with other studies that have reported an EDAN effect for gaze cues (Feng and Zhang 2014; Lassalle and Itier 2013). Arrows were chosen as a control stimulus to determine whether group differences were specific to gaze cues or more reflective of a general deficit in visuo-spatial orienting. This is particularly important given previous evidence of visuo-spatial orienting impairments in ADHD (Casagrande et al. 2012; Ortega et al. 2013).

Previous research has indicated that the processing of faces appears atypical in ASD and that this manifests in reduced amplitude, increased latency and/or altered topography of the N170 (Hileman et al. 2011; McPartland et al. 2011; Webb et al. 2006, 2012), an ERP thought to index face processing. One previous study reported reduced N170 right hemisphere lateralisation in children with ADHD but only if they had comorbid ASD (Tye et al. 2013). In this study, the authors used a face inversion paradigm with faces showing direct or averted gaze. We aimed to measure face processing in a directional gaze cueing paradigm to determine whether these findings are replicated in a different context. To achieve this, a face stimulus was presented immediately prior to the directional cue in the gaze cue condition and N170 amplitude was measured to the face stimulus over left and right occipital electrodes.

Based on evidence of the primacy of social cues in capturing and orienting attention (Johnson et al. 2005), we predicted that in typically developing children the size of the cue validity effect on target reaction time (RT) (shorter RT to targets cued by valid than invalid directional cues) and the EDAN effect (amplitude greater over occipital electrodes contralateral than ipsilateral to cue direction) would be larger for gaze than arrow cues. We further predicted that the N170 to the face stimulus would show right hemisphere lateralisation as found previously (Tye et al. 2013; Webb et al. 2006). We compared these effects between children with ADHD, ASD and comorbid ADHD+ASD and controls, treating ADHD and ASD as between-subjects factors. Specifically, we measured the main effect of ASD diagnosis by comparing those with ASD (ASD, ADHD+ASD) and without (CTRL, ADHD) and the effect of ADHD by comparing those with ADHD (ADHD, ADHD+ASD groups) and without (CTRL, ASD groups). First, we examined main effects of ADHD and ASD to establish whether alterations in gaze cueing and/or face processing are specific to ASD or also occur in ADHD. We predicted that if children with ADHD have an equivalent deficit in social cognition to those with ASD, there will be significant main effects of both ADHD and ASD; that is, having either diagnosis, or both, leads to atypical orienting of attention to gaze cues and altered face processing. This would suggest that any deficits in those with comorbid ADHD+ASD arise from additive effects of impairment in ADHD and ASD. Conversely, if impairments in ADHD occur only when comorbid ASD symptoms are present, the main effect of ASD will be significant but the main effect ADHD will be non-significant. This would suggest that impaired face and gaze processing are specific to ASD and that the comorbid ASD symptoms in those with ADHD reflect true overlap between these conditions at the neural systems level. Alternatively, if comorbid ASD symptoms in those with ADHD reflect only a phenocopy of ASD symptoms, the ASD group will differ from both the ADHD and ADHD+ASD groups. This would be reflected in an interaction between the ASD and ADHD factors. An interaction could also arise if the comorbid group show an entirely different pattern of effects from both the ADHD and ASD groups, which would suggest that the effect of having both conditions is not simply additive. We also examined linear relationships between dimensional measures of ASD and ADHD symptoms and the ERP measures described above.

## Methods

### Participants

Participants were 35 children aged 8–15 years diagnosed with or undergoing assessment for ASD or ADHD and 20 typically developing children recruited from the local community (Control Group; CTRL). The study procedures (including gaining informed consent) were approved by the United Kingdom National Research Ethics Committee. Children in the ASD/ADHD group were referred to the study by child and adolescent psychiatrists and community paediatricians or were identified from local support groups. To obtain a research diagnosis, each participant was assessed using the Development and Well-being Assessment (DAWBA; Goodman et al. 2000). Consensus DSM-IV diagnosis was reached between two child and adolescent psychiatrists (PK, CH). Participants in the clinical group were invited to attend a further assessment using the Autism Diagnostic Observation Schedule (ADOS; Gotham et al. 2006). Of 35 invited from the entire clinical sample (ASD and ADHD), 16 (46%) consented. Autism diagnosis was confirmed in 11 of these cases (10 had been referred to the study with ASD diagnosis or with ADHD plus ASD features; 1 was referred with ADHD only) and ruled out in the remaining five cases (all had been referred with ADHD diagnosis). Participants were assigned to the comorbid ADHD+ASD group if the DAWBA or ADOS indicated comorbid diagnosis. Otherwise they remained in the ‘ADHD’ or ‘ASD’ group (see Table 1). The presence/absence of ASD was established by combining information from the DAWBA, rating scales and ADOS in clinical consensus case reviews held by PK and CH.

**Table 1:** Group demographic and clinical characteristics

	CTRL (n = 20)	ADHD (n = 12)	ASD (n = 10)	ADHD+ASD (n = 13)	Group comparisons	
Age	12.58 (1.92)	11.94 (2.35)	12.52 (1.76)	12.51 (2.36)	ns	
Gender ratio (%male)	80	53	90	92.3	ns	
IQ (WASI)	111.35 (8.85)	97.00 (17.06)	106.00 (8.52)	95.54 (16.87)	ADHD+ASD < CTRL	
SCQ total	2.78 (2.02)	9.58 (6.83)	21.40 (8.90)	23.31 (7.69)	CTRL < All; ASD > ADHD, ADHD+ASD > ADHD	
RSI	0.89 (0.83)	3.33 (2.71)	8.20 (4.71)	9.10 (3.35)		
SC	1.44 (1.10)	2.92 (2.07)	6.70 (3.16)	7.38 (2.36)		
RRBI	0.33 (0.69)	2.50 (2.65)	4.80 (2.39)	5.15 (2.41)		
Conners ADHD-index	45.89 (5.34)	79.33 (11.00)	67.70 (11.83)	79.69 (5.04)	CTRL < All; ADHD > ASD, ADHD+ASD > ASD	
Comorbid diagnoses (n per group)						
Dep/Anxiety	0	2	1	5		
CD/ODD	0	7	5	6		
OCD	0	0	0	1		

*WASI* Wechsler abbreviated scale of intelligence, *SCQ* social communication questionnaire, *RSI* restricted social interaction sub-scale of SCQ, *SC* social communication sub-scale, *RRBI* restrictive, repetitive behaviors sub-scale of SCQ, *CD/ODD* conduct disorder/oppositional defiant disorder, *OCD* obsessive compulsive disorder

ASD symptom severity was measured using the lifetime parent-rated version of the Social Communication Questionnaire (SCQ; Rutter et al. 2003) and ADHD symptom severity was measured using the ADHD Index of the long version of the Conners Parent Rating Scale (CPRS; Conners 1997). The SCQ Total Score and three sub-scales [Restricted Social Interaction (‘RSI’), impaired Social Communication (‘SC’) and Restricted and Repetitive Behaviours and Interests (‘RRBI’)] were computed.

Exclusion criteria were intellectual disability [determined by clinical diagnosis or IQ < 70 on the Wechsler Abbreviated Scale of Intelligence (Wechsler 1999)], Tourette Syndrome/chronic tic disorder or other significant neurological/genetic disability. Children in receipt of non-stimulant medication for ADHD were not recruited as these long-acting medications cannot be withdrawn temporarily. Of 168 children referred, 20 stated they did not wish to take part and 90 initially expressed an interest but later withdrew or were uncontactable. Of the remaining 58, 17 met an exclusion criterion (outside age range n = 6; no confirmed/consensus diagnosis of ADHD or ASD n = 3; tic disorder n = 3; IQ < 70 n = 3; non-stimulant medication n = 3). Of 40 eligible participants, five withdrew from the study after consenting, leaving a sample of 35.

Controls were recruited from local schools and a database of healthy volunteers held by the School of Psychology, University Of Nottingham. Exclusion criteria for the typical sample were score >65 on the Conners ADHD index and >15 on the SCQ, indicative of risk of ADHD and ASD respectively, in addition to those listed above. Of 32 children whose parents responded to the study information, 8 decided not to take part after receiving further information, 1 was deemed ineligible (Kleinfelter Syndrome) and 1 scored above threshold on the SCQ. Of the remaining 22, 1 was not available for testing within the time-frame the study and EEG data were not recorded for 1 participant due to a technical fault, leaving a sample of 20.

Other mental health/behavioural problems (depression/anxiety, obsessive compulsive disorder (OCD), oppositional defiant disorder (ODD) and Conduct Disorder (CD)) were not excluded (see Table 1).

### Experimental Task

The experiment was programmed in E-Prime version 1.2 (Psychology Software Tools, Pittsburgh, PA) presented on a 17-inch computer monitor positioned 60 cm in front of the participant. Manual button-press responses were recorded using a Cedrus Superlab button box (Cedrus Corporation, San Pedro, CA).

**Fig. 1:**
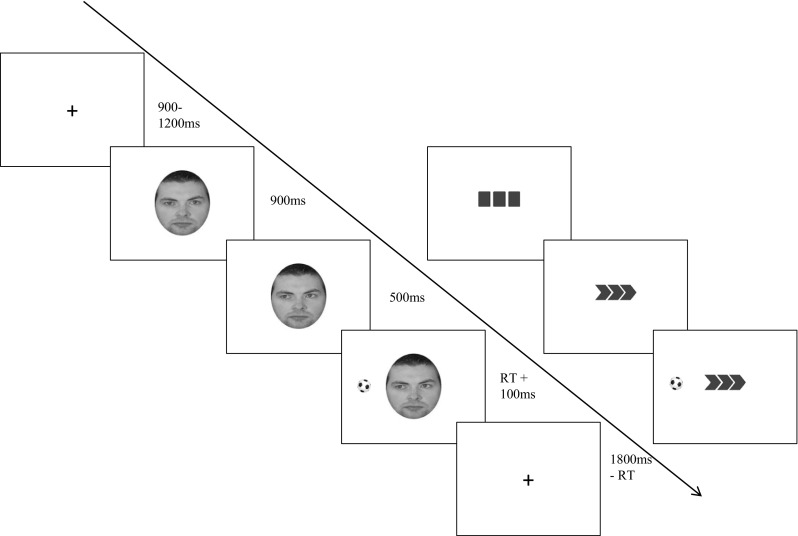
The figure shows the structure of gaze and chevron cueing trials in the visuo-spatial attention cueing task. Trial length is indicated by the *arrow* with stimulus durations shown in milliseconds. The left side of the diagram shows an example of a gaze cueing trial in which cue direction and target location are congruent. The right-hand side shows a chevron-cueing trial in which cue direction and target location are incongruent

Stimuli for the gaze cue condition comprised a photographic image of an adult male face presented within an oval measuring 42 mm height by 35 mm width, on a white background (Fig. 1). Each eye measured 3 mm height by 7 mm width. To create the directional gaze cue the iris and pupil within each eye were repositioned (using Adobe Photoshop) such that they appeared to move from a central (direct) gaze to a leftward or rightward gaze. Once repositioned, the pupil was 3 mm to the left or right of the central point. The face stimulus was partially occluded so that the forehead and hairline were visible but ears and neck were not. This ensured the eyes were the focal part of the image and were sufficiently large on the screen to act as the cue.

In the arrow (non-social) condition the directional cue comprised a set of three chevrons pointing to the left or right of fixation (created in Microsoft Office); each measured 5 mm at their widest point and 8 mm high, spaced 2 mm apart. Immediately preceding the onset of the directional cue, three blocks were presented so that the appearance of the chevrons led to a sense of movement equivalent to the movement of the eyes in the gaze cue condition. In both conditions the target was a black and white football measuring 15 mm diameter, presented to the left or right of cue centre at a distance of 75 mm.

On each trial, a fixation cross presented for 900–1200 ms (jittered) was replaced by either a face (gaze condition) or three blocks (arrow condition) for 800 ms. This was followed by the directional cue (gaze or chevron). The target appeared after 500 ms and both cue and target remained on-screen until a response was made. The inter-trial interval was adjusted for all subjects based on the length of time between target-onset and response, to provide a uniform trial length within- and between-subjects. Cue direction was either a valid or invalid predictor of target location. The ratio of valid:invalid trials was 50:50. Cue validity was randomised between trials.

The task was presented in 20 blocks; 10 gaze and 10 chevron. Each block comprised 20 trials from the same condition. Conditions were alternated and order was counterbalanced between-subjects. Median RT and proportion of directional errors were computed.

### Electrophysiological Data Recording

EEG data were recorded at a sampling rate of 1024 Hz using a Biosemi Active II system (Biosemi, Netherlands) with 128 silver/silver chloride (Ag/AgCl) electrodes positioned according to an extended 10–20 montage. During recording signals were referenced to a common mode sensor to the left of Cz. Additional electrodes were placed at the inner orbital ridge and the outer canthus of each eye to record eye movements and on the left and right mastoid process to record other artefacts.

### Electrophysiological Data Processing and Analysis

Data analysis was performed using EEGLab version 13.05.04b (Delorme and Makeig 2004) run on Matlab version 8.4. Channels with extreme, high amplitude noise (>500 µV over more than 50% of the dataset) were rejected and the data were re-referenced to the average of the remaining channels before filtering between 0.5 and 45 Hz decibels/octave. Next, data were visually inspected to identify and reject periods of extreme noise spanning all channels. Long epochs (−1000 to 2000 ms) time-locked to the initial alerting stimulus (face/blocks) were extracted and extended infomax Independent Component Analysis (ICA) was performed to identify artefacts using the ‘Semi-Automated Selection of Independent Components of the electroencephalogram for Artifact correction’ (SASICA) toolbox (Chaumon et al. 2015). After rejecting artefactual components, weights of the remaining components were back-projected onto the original data. Noisy channels removed prior to ICA were then interpolated using the spherical spline function in EEGLab. The data were segmented into epochs in two ways: firstly, to measure the EDAN, peri-stimulus epochs −200 to 500 ms locked to the directional (leftward, rightward) cue were extracted; secondly, to measure the N170, epochs of −200 to 800 ms locked to the face stimulus were extracted. Baseline correction was performed using the 200 ms pre-stimulus baseline period. Finally, trials were averaged to create single subject ERP waveforms for the face stimulus and for each cue direction and cue type.

The Early Directing Attention Negativity (EDAN) was computed at occipital electrodes O1 (left hemisphere) and O2 (right hemisphere) as mean amplitude within a 200–300 ms time window for each Cue Type (Chevron, Gaze) and Cue Direction (Leftward, Rightward). The N170 to the Face stimulus was defined as maximal negative amplitude in the 150–200 ms post-stimulus time window at the lateral occipital electrodes, O1 and O2. Peak detection was performed semi-automatically (i.e. the experimenter visually inspected the peaks selected automatically by the software). When the peak fell just outside the pre-defined window, the experimenter identified the correct peak manually. If no discernible peak was identified, the peak marker was moved to the centre of the window. The time windows for both ERPs were chosen according to previous studies in children (Shimi et al. 2014; Tye et al. 2013).

### Procedure

In the first phase of the study the DAWBA was conducted with a parent of each participant, either in person or online. Parents also completed the Conners, SCQ and a measure of socio-economic status (Office for National Statistics 2004). The child completed the WASI at this stage if there were concerns about intellectual function; otherwise this was completed immediately prior to EEG. After set-up of the EEG, children performed a short practice comprising ten example trials from the experimental task. The task was readily understood by all children after one or two practice tasks. The main task lasted roughly 35 min including rest breaks. After completion, resting state data (not reported here) were recorded for approximately 5 min. Participants were then de-briefed and awarded an inconvenience allowance. Participants in the clinical group were invited to take part in the ADOS and those who did so were paid an additional allowance.

### Statistical Analysis

Factorial ANOVAs were employed comprising two between-subjects factors, ASD and ADHD, each with two levels ‘Absent’ and ‘Present’. Additional within-subjects factors are described in more detail below.

#### Target RT

After removing short (<100 ms) and long (>1200 ms) values, RT data were significantly skewed. A log-_10_ transform normalised the distribution and the transformed variables were entered into mixed design ANOVA with the between-subject factors described above and three within-subjects factors: Cue Type (Chevron, Gaze), Target Visual Field (Left, Right) and Cue Validity (Valid, Invalid).

#### EDAN Amplitude to Directional Cues

To compute the typical EDAN effect (greater amplitude over contralateral than ipsilateral hemisphere), mean amplitude across electrodes ipsilateral to cue direction for leftward and rightward cues and mean amplitude across contralateral electrodes for leftward and rightward cues, were computed for each cue type. This method provided a measure of ipsilateral and contralateral amplitudes collapsed across cue direction. ANOVA was performed with two within-subjects factors, Cue Type (Chevron, Gaze) and Hemisphere (Ipsilateral, Contralateral) in addition to the between-subjects factors described above.

#### N170 to the Face Stimulus

N170 amplitude was entered into ANOVA with one within-subjects factor, Hemisphere, with two levels (Left, Right) in addition to the between-subjects factors described above.

All main effects and interactions significant at p < .05 were followed up with appropriate simple effects analysis.

#### Correlational Analyses between ERPs and Symptom Scores

Dimensional analyses were performed by calculating correlations between SCQ Total scores and ERP measures that were found to be significantly predicted by either the ASD or ADHD factors in the factorial ANOVAs described above. Correlations were also computed between each ERP measure and Conners ADHD-Index. Bonferroni correction for multiple comparisons was computed separately for each set of correlations (described in more detail in the “Results” section). When a significant correlation was identified between SCQ Total scores and an ERP variable, further correlations were computed between the SCQ sub-scales (RSI, SC, RRBI) to determine which aspects of ASD symptomatology were driving the correlation.

Finally, the possible influences of IQ (which differed significantly between groups, see Table 1) and age (to control for general developmental effects on the behavioural and ERP measures) were examined by re-running all models described above with IQ included as a covariate in ANCOVA and then with age included as a covariate. The results are reported only if either covariate was a significant predictor or if the pattern of effects was altered by adding in these covariates.

## Results

### Performance

Group mean error rates were below <7% in all groups: CTRL group mean = 1.66%, SD = 1.42; ASD group mean = 1.63, SD = 1.57; ADHD group mean = 6.98%, SD = 6.59; ADHD+ASD group mean = 4.63, SD = 6.43.

ANOVAs were performed on the log-transformed median RT variables (descriptive statistics are reported in Table 2 for the original non-transformed variables for ease of interpretation). There was a main effect of Cue Validity (Valid < Invalid; *F* (1, 51) = 36.07, *p* = .000, η_p_^2^ = 0.41). There was also a main effect of ADHD on RT (*F* (1, 51) = 8.51, *p* = .005, η_p_^2^ = 0.14) with significantly longer RTs for ADHD Present than Absent. ADHD interacted with Cue Validity (*F* (1, 51) = 6.01, *p* = .018, η_p_^2^ = 0.11) with a larger validity effect for ADHD Present (*F* (1, 51) = 34.61, *p* = .000, η_p_^2^ = 0.40) than Absent (*F* (1, 51) = 6.54, *p* = .014, η_p_^2^ = 0.11). This was due to a greater difference in RT between invalid and valid trials in ADHD Present than Absent. In addition, the difference in RT between ADHD Present and Absent was numerically greater for invalid trials than valid trials. Finally, there was a significant interaction between Target VF and Cue Validity (*F* (1, 51) = 4.40, *p* = .041, η_p_^2^ = 0.08), with longer RT for left than right VF on valid trials (*F* (1, 51) = 8.48, *p* = .005, η_p_^2^ = 0.14) but no such difference on invalid trials (*F* (1, 51) = 0.29, *p* = .59, η_p_^2^ = 0.01). Neither the main effect of ADHD nor the ASD*ADHD interaction reached significance and these factors did not interact significantly with any other factors.

**Table 2:** Reaction time by group and trial type on visuo-spatial attention cueing task

	CTRL		ADHD		ASD		ADHD+ASD	
	Valid	Invalid	Valid	Invalid	Valid	Invalid	Valid	Invalid
Chevron								
Left VF	312.30 (11.98)	325.20 (15.78)	370.33 (19.80)	400.71 (29.39)	324.20 (12.15)	335.65 (13.06)	369.04 (19.40)	392.00 (23.70)
Right VF	313.98 (14.07)	321.78 (16.52)	375.75 (24.18)	421.63 (35.86)	313.10 (11.64)	334.50 (16.78)	352.65 (14.52)	379.27 (22.99)
Gaze								
Left VF	320.33 (13.14)	325.60 (16.45)	375.79 (28.09)	405.08 (29.74)	329.75 (10.94)	338.65 (12.00)	367.12 (17.77)	388.77 (23.96)
Right VF	315.33 (14.48)	325.45 (17.76)	361.25 (26.42)	412.63 (34.60)	319.45 (13.44)	335.60 (14.12)	349.88 (14.05)	375.62 (18.94)

Data are group means with SD in parentheses

‘Valid’ refers to trials on which the cue was a valid predictor of target location; ‘Invalid’ refers to trials on which the cue was not a valid predictor of target location

*VF* visual field

With IQ included in the model, the ADHD by Validity interaction no longer reached significance (*F* (1, 50) = 2.73, *p* = .11, η_p_^2^ = 0.05); the pattern of significant and non-significant effects remained unchanged apart from this and IQ was not a significant independent predictor. With age included in the model, the ADHD by Validity interaction remained significant (*F* (1. 50) = 5.52, *p* < .05, η_p_^2^ = 0.1) and no other effects changed. Age was a significant predictor of RT overall (*F* (1, 50) = 8.17, *p* < .01, η_p_^2^ = 0.14).

### EDAN Amplitude to Directional Cues

**Fig. 2:**
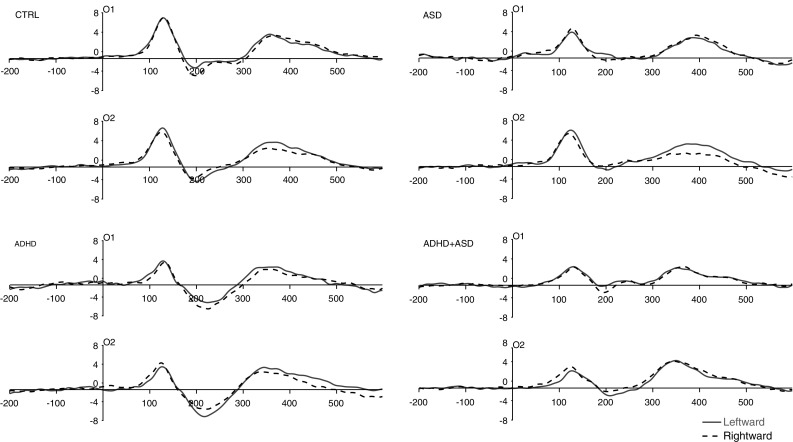
Each plot shows ERP waveforms for the chevron cue at electrodes O1 (*left* occipital hemisphere) and O2 (*right* occipital hemisphere) for leftward (*solid line*) and rightward (*dashed line*) cue trials in each group. Time is shown on the *x-*axis in milliseconds. Amplitude is shown on the *y-*axis in microvolts. Stimulus-onset is shown at time ‘0’

**Fig. 3:**
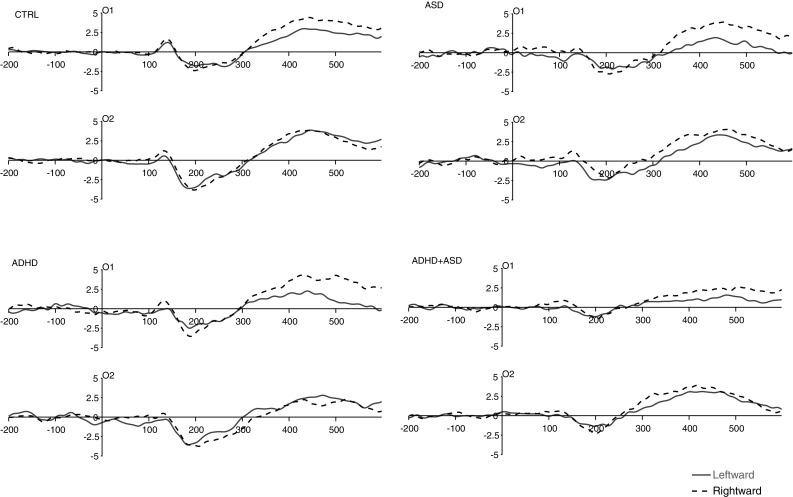
Each plot shows ERP waveforms for the gaze cue at electrodes O1 (*left* occipital hemisphere) and O2 (*right* occipital hemisphere) for leftward (*solid line*) and rightward (*dashed line*) cue trials in each group. Time is shown on the *x-*axis in milliseconds. Amplitude is shown on the *y-*axis in microvolts. Stimulus-onset is shown at time ‘0’

**Fig. 4:**
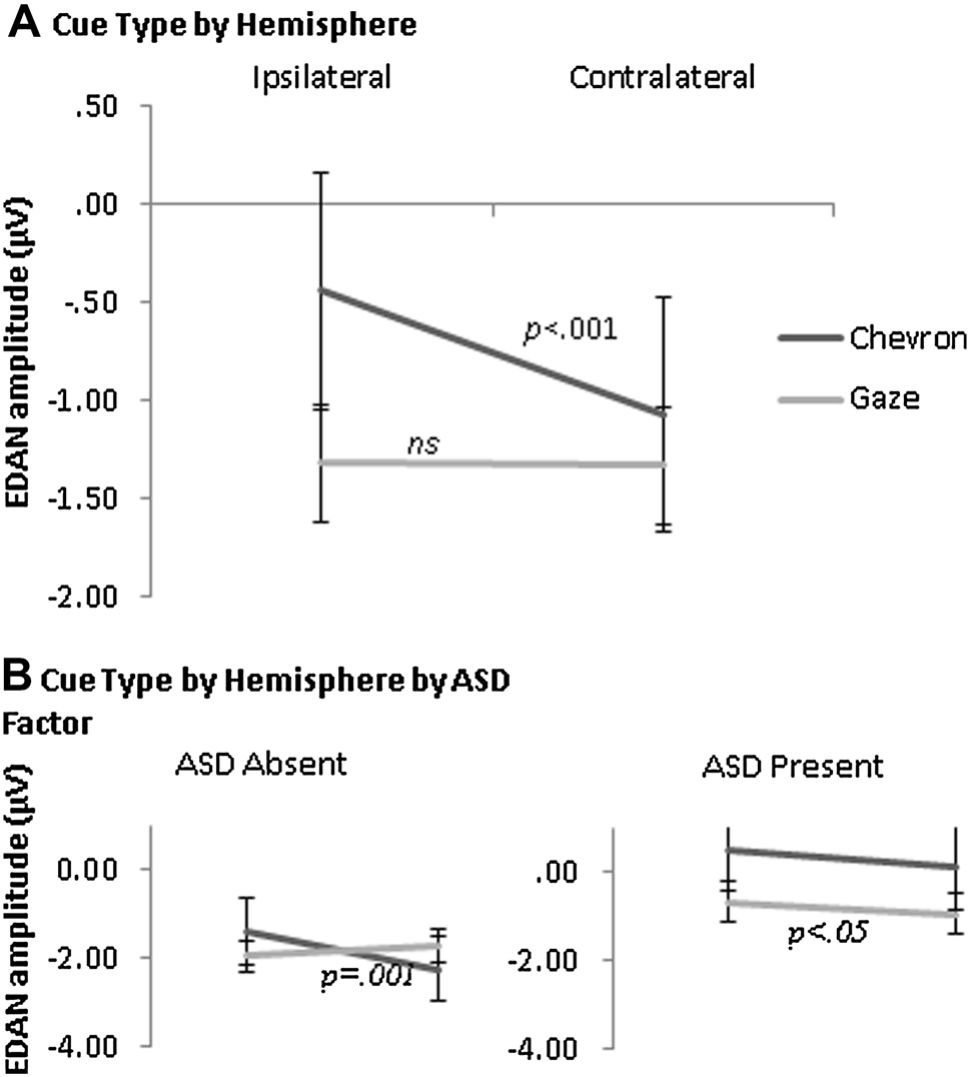
The interaction between Cue Type and Hemisphere on EDAN amplitude is shown in** a** and the interaction between Cue Type, Hemisphere and ASD is shown in** b** with ASD Absent (CTRL, ADHD) shown on the *left* and ASD Present (ASD, ADHD+ASD) shown on the *right. Lines* show adjusted means with* standard error* of the mean. EDAN amplitude is shown on the *y*-axis in microvolts

ERP waveforms for directional chevron and gaze cues are shown in Figs. 2 and 3 respectively. As predicted, amplitude was significantly greater over Contralateral than Ipsilateral hemisphere (Main effect Hemisphere: *F* (1, 51) = 9.73, *p* = .003, η_p_^2^ = 0.16) confirming the typical EDAN effect. This was qualified by a two-way Cue Type by Hemisphere interaction (*F* (1, 51) = 10.65, *p* = .002, η_p_^2^ = 0.17): amplitude was significantly greater over contralateral than ipsilateral hemisphere for Chevron cues (*F* (1, 51) = 16.78, *p* = .000, η_p_^2^ = 0.25), but not Gaze cues (*F* (1, 51) = 0.01, *p* = .914, η_p_^2^ = 0.00) (see Fig. 4). This was further qualified by a three-way interaction between Cue Type, Hemisphere and ASD factor (*F* (1, 51) = 6.71, *p* = .012, η_p_^2^ = 0.12): ASD Absent showed a Cue Type by Hemisphere interaction (*F* (1, 51) = 15.59, *p* = .000, η_p_^2^ = 0.34) with significantly greater contralateral than ipsilateral amplitude for Chevron cues (*F* (1, 51) = 14.80, *p* = .001, η_p_^2^ = 0.33) but no effect for Gaze cues (*F* (1, 51) = 2.29, *p* = .14, η_p_^2^ = 0.07). Conversely, ASD Present showed no Cue Type by Hemisphere interaction (*F* (1, 51) = 0.09, *p* = .57, η_p_^2^ = 0.02) but a main effect Hemisphere (*F* (1, 51) = 4.84, *p* = .039, η_p_^2^ = 0.19) (contralateral > ipsilateral) indicating an equivalent EDAN effect for both cue types (Fig. 3). In addition, ASD was a near-significant predictor of amplitude overall (*F* (1, 51) = 3.92, *p* = .053, η_p_^2^ = 0.07) with smaller amplitude in ASD Present than Absent. Neither the main effect of ADHD nor the ASD by ADHD interaction reached significance and these factors did not interact significantly with any other factors. Neither IQ nor age were significant predictors and neither altered the pattern of effects reported.

**Fig. 5:**
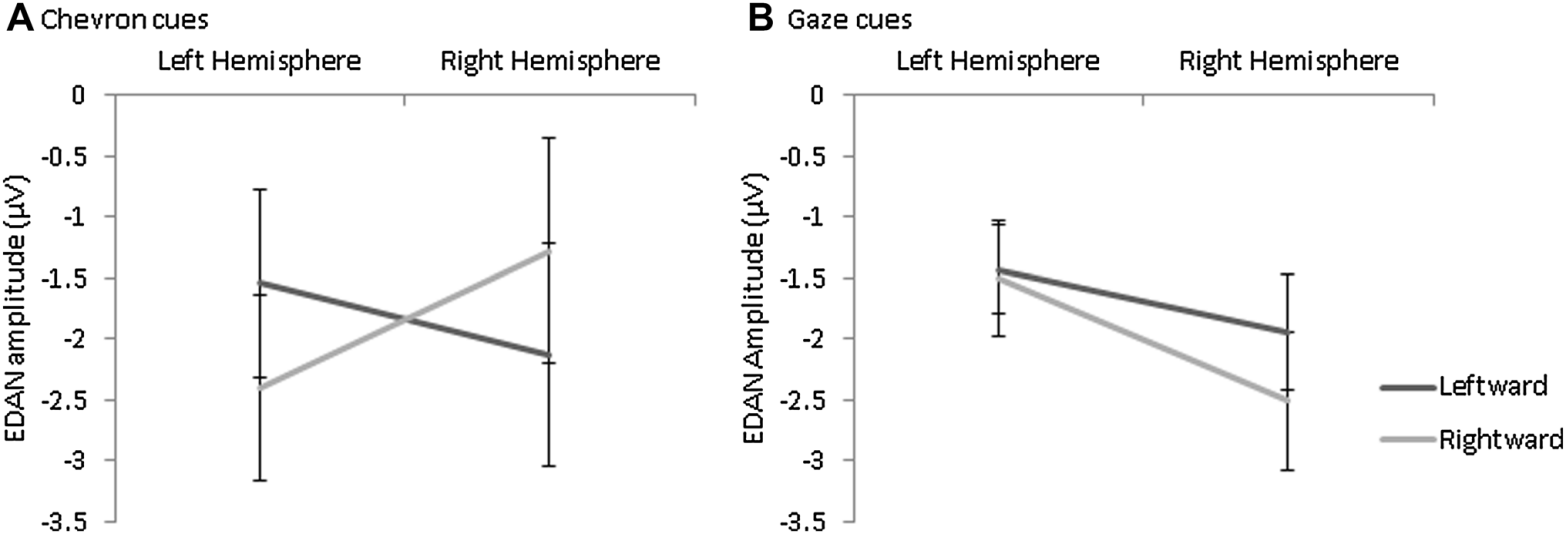
The significant interaction between Cue Direction and Electrode hemisphere is shown for Chevron cues (**a**) and the main effect of Electrode hemisphere is shown for Gaze cues (**b**) in the ASD Absent group (CTRL, ADHD). *Lines* show adjusted means with standard error of the mean. EDAN amplitude is shown on the *y*-axis in microvolts

To determine whether the difference in EDAN effect between cue types in the ASD Absent group (CTRL, ADHD) was specific to one electrode or cue direction, an additional ANOVA was carried out with the factors Cue Type, Cue Direction (Leftward, Rightward) and Electrode (Left Hemisphere, Right Hemisphere) on ASD-Absent participants only. The ADHD Factor was also included but did not interact significantly with the ASD factor or with any within-subjects factors. There were significant interactions between Cue Type and Electrode (*F* (1, 51) = 5.70, *p* = .024, η_p_^2^ = 0.16), Cue Direction and Electrode (*F* (1, 51) = 5.01, *p* = .033, η_p_^2^ = 0.14) and between Cue Type, Cue Direction and Electrode (*F* (1, 51) = 15.59, *p* = .000, η_p_^2^ = 0.34) (See Fig. 5). Simple effects were investigated first by analysing the effects of Cue Direction and Electrode at each level of Cue Type. There was a Cue Direction by Electrode interaction for Chevron (*F* (1, 51) = 14.80, *p* = .001, η_p_^2^ = 0.33) but not Gaze (*F* (1, 51) = 2.29, *p* = .14, η_p_^2^ = 0.07), replicating the Cue Type by Hemisphere interaction identified in the initial ANOVA reported above. Secondly, the effects of Cue Type and Cue Direction were analysed at each level of Electrode. At the left hemisphere electrode there was a trend towards a main effect of Cue Direction (*F* (1, 51) = 3.51, *p* = .07, η_p_^2^ = 0.11) with greater amplitude for rightward than leftward cues. At the right hemisphere electrode, there was a significant Cue Type by Cue Direction interaction (*F* (1, 51) = 16.57, *p* = .000, η_p_^2^ = 0.36): for the Chevron cue, amplitude was greater for leftward than rightward cues (*F* (1, 51) = 7.44, *p* = .01, η_p_^2^ = 0.20), consistent with the EDAN effect. For Gaze cues however, amplitude was significantly greater for rightward than leftward cues (*F* (1, 51) = 5.48, *p* = .026, η_p_^2^ = 0.15), opposite to the typical EDAN effect.

### N170 to the Face Stimulus

**Fig. 6:**
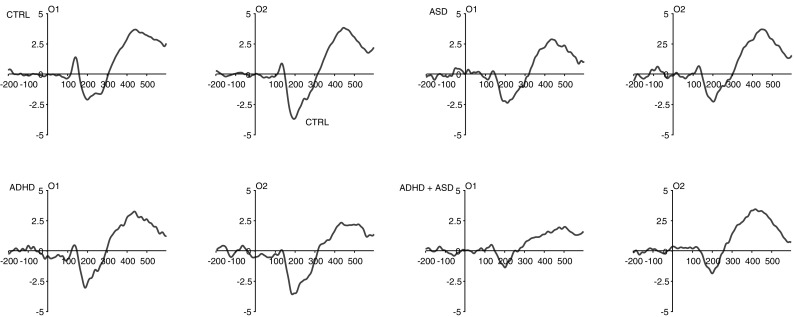
Each plot shows group averaged ERP waveforms for the face stimulus at electrodes O1 (*left* occipital hemisphere) and O2 (*right* occipital hemisphere). Time is shown on the *x*-axis in milliseconds. Amplitude is shown on the *y*-axis in microvolts. Stimulus-onset is shown at time ‘0’

The ASD factor was a significant predictor of N170 amplitude (*F* (1, 51) = 10.11, *p* = .003, η_p_^2^ = 0.17) with smaller amplitude in ASD Present than Absent (see Fig. 6). In addition, ASD interacted with Site at a marginal level of significance (*F* (1, 51) = 3.64, *p* = .06, η_p_^2^ = 0.07). Although not quite reaching significance, this predicted effect was explored by analysing the effect of Site at each level of ASD: for ASD Absent, there was significantly greater amplitude over right than left hemisphere (*F* (1, 51) = 5.48, *p* = .02, η_p_^2^ = 0.10) but for ASD Present this difference was non-significant (*F* (1, 51) = 0.24, *p* = .62, η_p_^2^ = 0.01). The interaction also manifested in significantly greater amplitude in ASD Absent than ASD Present over right hemisphere (*F* (1, 51) = 13.57, *p* = .001, η_p_^2^ = 0.21) but only a trend-level difference over left hemisphere (*F* (1, 51) = 3.66, *p* = .06, η_p_^2^ = 0.07). The main effect of ADHD and the ASD by ADHD interaction were not significant and these terms did not interact with any other factors. Neither IQ nor age were significant predictors and neither altered the pattern of effects reported above.

### Correlational Analysis between ERPs and Symptom Scores

Correlations were computed between SCQ Total and EDAN amplitude to gaze cues for each cue direction and electrode hemisphere (four ERP variables in total). The correlation was significant only for rightward cues at the right hemisphere electrode (r = .30, *p* = .03) with smaller (more positive) amplitude in those with higher SCQ scores. This effect did not survive correction for multiple comparisons (Bonferroni-corrected alpha of 0.013 to control Type 1 error rates arising from four pairwise correlations between SCQ Total and each ERP amplitude). N170 amplitude over right (but not left) hemisphere correlated significantly with SCQ Total scores (r = .35, *p* = .01) which survived Bonferroni correction (critical alpha 0.025 based on two pairwise correlations between SCQ Total and N170 amplitude at each electrode). Further analysis was performed to determine which sub-scales from the SCQ correlated with N170 amplitude over the right hemisphere. The SC (r = .32, *p* = .02) and RSI (r = .42, *p* = .002) sub-scales correlated significantly but not the SI subscale. None of the amplitude measures correlated significantly with the Conners ADHD-index.

## Discussion

This study compared children with ASD, ADHD or ADHD+ASD and typically developing controls on behavioural and electrophysiological correlates of attentional orienting and face processing. We measured effects of ASD, ADHD and their interaction on the EDAN, an ERP marker of the orienting of visual attention towards a spatially cued location and the N170, a right-hemisphere lateralised ERP linked to face processing. We identified atypical gaze cue and face processing in children with ASD and ADHD+ASD, suggesting that these ERPs may reflect neurobiological mechanisms underlying the overlap between ADHD and ASD.

Firstly, there was a significant EDAN effect (greater amplitude over contralateral than ipsilateral occipital electrodes) for Chevron but not Gaze cues, replicating the findings of previous gaze cueing studies that have reported a lack of EDAN for gaze cues (Brignani et al. 2009; Hietanen et al. 2008; Holmes et al. 2010). Interestingly however, this difference between cue types was unique to the ASD-Absent groups (CTRL, ADHD). Further analysis of this pattern revealed that in the ASD-Absent group, there was a profile of greater amplitude over right hemisphere for Gaze cues irrespective of cue direction while for Chevron cues, the typical EDAN effect was identified. In contrast, the ASD-Present group (ASD, ADHD+ASD) showed equivalent EDAN for both cue types. This unpredicted effect requires replication in a new sample before drawing firm conclusions. However, one tentative interpretation is that in the ASD-Absent group, the typical contralateral > ipsilateral EDAN effect for gaze cues is obscured by additional right-hemisphere specific processing of gaze. This is consistent with previous evidence that gaze cue processing is right-hemisphere dominant (Akiyama et al. 2006; Greene and Zaidel 2011). Conversely, children with ASD showed the typical EDAN effect for Gaze and Chevron cues, suggesting an absence of this right-hemisphere specialisation. This interpretation is further substantiated by the correlational analysis showing weaker EDAN amplitude to Gaze cues specific to the right hemisphere electrode in those with higher SCQ scores (although it is important to note that this correlation did not survive Bonferroni correction for multiple comparisons). Moreover, there were no effects of ASD on RT to gaze cues and error rates were low across all groups. This is consistent with previous research (reviewed in Landry and Parker 2013) and suggests that children with ASD can perform simple social cognition tasks equivalently to their age- and gender-matched typical peers, but they may use different neural systems to achieve this (Greene et al. 2011).

Secondly, there was reduced right hemisphere lateralisation of the N170 in those with ASD (ASD, ADHD+ASD), replicating the findings of Tye et al. (2013) in a different experimental task. These findings and the EDAN effects described above add to evidence of altered neural correlates of gaze cue and face processing in ASD (Nomi and Uddin 2015) and further suggest that this atypical processing is found in those with comorbid ADHD+ASD. This is important because it provides some preliminary evidence of brain mechanisms that might partly explain the overlap between ASD and ADHD and supports the assertion that the co-occurrence between the two is not due to phenocopy (when symptoms of one condition mimic those of another) (Rommelse et al. 2011). Moreover, the null effect for the interaction between ADHD and ASD coupled with main effects for ASD indicates that the ADHD+ASD group are statistically equivalent to the ASD group. These findings suggest that interventions to target social cognition may be appropriate for comorbid ADHD+ASD, although caution is needed given that ADHD symptoms may undermine social skills interventions in comorbid cases (Davis and Kollins 2012). Indeed, in the present study, ADHD was associated with longer RTs overall and a greater cue validity effect, arising from longer RTs to invalidly cued targets in ADHD-Present (ADHD, ADHD+ASD) than Absent (ASD, CTRL). This effect did not interact with Cue Type, suggesting a general attentional deficit rather than impaired social cognition. This attentional deficit could undermine attempts to engage in social-skills interventions designed to enhance social function in those with autism and could explain why these interventions are less successful in children with autism who have comorbid ADHD (Davis and Kollins 2012).

At the behavioural level, there were no significant differences in RT between gaze and chevron cues in any group. This conflicts with previous studies showing larger validity effects to gaze than arrow cues (reviewed in Frischen et al. 2007). This could be because in the present study the task design was optimised for measuring ERPs and therefore used an SOA of 500 ms. In earlier studies, gaze cues primarily influence the reflexive orienting of attention captured at short SOAs (Frischen et al. 2007). Arguably however, the unique and rapid effects of gaze cues on attention are captured in the EDAN rather than performance measures in the present study and so the findings are broadly consistent with prior research. Similarly, although we did not replicate some previous studies reporting a stronger RT validity effect in the left than right visual field for gaze cues (e.g. Greene and Zaidel 2011; Marotta et al. 2012), the EDAN effect over the right hemisphere (described above) is consistent with right hemisphere specialisation for gaze cue processing.

The present study did not find any evidence of performance deficits when analysing response times to gaze cues in children with ASD. Although this may be due to the small sample sizes in this study, behavioural effects in ASD have often not been replicated (see Landry and Parker 2013; Nation and Penny 2008). This could reflect compensatory mechanisms that facilitate typical performance in some individuals with ASD. The findings of the present study, showing typical performance but with an altered neural profile, are consistent with this. Nevertheless, further research using larger, carefully defined samples, is needed to explore the influence of factors such as intellectual ability, age and symptom severity to fully understand the reasons why ASD-related impairments in orienting attention to gaze cues are not always found, particularly given the obvious impairments in joint attention and social referencing in the everyday functioning of these individuals. This study also failed to replicate the findings of Marotta et al. (2014) showing a reduced gaze orienting effect in ADHD. This could be because the effects in the ADHD group in the Marotta et al., study were driven by sub-threshold ASD symptoms. Alternatively, differences in task design, sample sizes and other methodological features could explain these between-study differences.

The factorial approach used here increased the power to identify main effects of ASD and ADHD, despite small sample sizes for the individual diagnostic groups. There was sufficient power (96%) to detect at least a medium effect size for the interaction between the ADHD and ASD factors, at an alpha criterion of 0.05. This suggests that if an interaction between the ADHD and ASD factors exists, it is likely to be smaller than a medium effect and is also likely to be smaller than the main effects of ASD reported above. However, given the small sample sizes in this study, further research is needed to replicate the findings and to explore the influence of other conditions such as CD, ODD and mood/anxiety disorders that are frequently comorbid with ADHD (Nijmeijer et al. 2008). In addition, less than half the cases in the clinical sample consented to the ADOS interview. Confirmation of research diagnoses with a parental semi-structured clinical interview, coupled with observation of the young person, is the gold standard. Some caution is therefore needed when interpreting the results of this study because the ADOS was not used in all cases. However, in those for whom ADOS data were available, the presence/absence of ASD was consistent with the diagnoses derived from DAWBA and clinical case review, suggesting that this process was reliable with respect to ASD. This method has also been used in recent epidemiological studies (Johnson et al. 2010). Despite this, ideally the study should be replicated in samples with a structured observation schedule. At this point, the findings should therefore be viewed as preliminary and as a starting point for a larger, well-powered study with carefully defined groups.

Further research is also needed to compare children with ASD and ADHD at an earlier point in development and on a range of measures of social cognition. Face and gaze processing develop early in infancy and so disruption at this stage of development may undermine the later development of more complex social cognition skills including joint attention (Johnson et al. 2005). Indeed, these more complex aspects of social cognition, such as theory of mind, empathy and emotion processing may be equivalently impaired in ADHD and ASD (Uekermann et al. 2010), although it remains to be established whether the pathways to impairment differ. A further point is that in this study we focussed on measures of social cognition and this is likely to be why the effects we found relate to ASD symptoms rather than ADHD symptoms. Had we manipulated other features of cognition such as inhibitory control or sustained attention that are often impaired in ADHD, we might have also identified effects that explained the presence of ADHD symptoms in the ASD group. Previous work has shown that children with ADHD+ASD show additive impairments that are found separately in both ADHD and ASD (Tye et al. 2013, 2014). A fuller exploration of the profile of impairments across a range of measures is therefore needed to gain a complete picture of ADHD and ASD comorbidity. Longitudinal measurements from early in childhood will further illuminate the time-course of these developmental abnormalities and the pathways to different syndromes, including comorbidity.
